# A world dataset on the geographic distributions of Solenidae razor clams (Mollusca: Bivalvia)

**DOI:** 10.3897/BDJ.7.e31375

**Published:** 2019-01-31

**Authors:** Hanieh Saeedi, Mark J Costello

**Affiliations:** 1 Department of Marine Zoology, Crustaceans, Senckenberg Research Institute and Natural History Museum , 60325 Frankfurt am Main, Germany Department of Marine Zoology, Crustaceans, Senckenberg Research Institute and Natural History Museum 60325 Frankfurt am Main Germany; 2 Institute for Ecology, Diversity and Evolution, Goethe University Frankfurt, Frankfurt am Main, Germany Institute for Ecology, Diversity and Evolution, Goethe University Frankfurt Frankfurt am Main Germany; 3 OBIS data manager, deep-sea node, Frankfurt am Main, Germany OBIS data manager, deep-sea node Frankfurt am Main Germany; 4 Institute of Marine Science, University of Auckland, Auckland 1142, New Zealand Institute of Marine Science, University of Auckland Auckland 1142 New Zealand

**Keywords:** Global, geographic distributions, Solenidae, razor clams, Mollusca, dataset, quality control, museum collections, latitudinal gradient in species richness.

## Abstract

**Background:**

Using this dataset, we examined the global geographical distributions of Solenidae species in relation to their endemicity, species richness and latitudinal ranges and then predicted their distributions under future climate change using species distribution modelling techniques (Saeedi et al. 2016a, Saeedi et al. 2016b). We found that the global latitudinal species richness in Solenidae is bi-modal, dipping at the equator most likely derived by high sea surface temperature (Saeedi et al. 2016b). We also found that most of the Solenidae species will shift their distribution ranges polewards due to global warming (Saeedi et al. 2016a). We also provided a comprehensive review of the taxon to test whether the latitudinal gradient in species richness was uni-modal with a peak in the tropics or northern hemisphere or asymmetric and bimodal as proposed previously (Chaudhary et al. 2016).

**New information:**

This paper presents an integrated global geographic distribution dataset for 77 Solenidae taxa, including 3,034 geographic distribution records. This dataset was compiled after a careful data-collection and cleaning procedure over four years. Data were collected using field sampling, literature and from open-access databases. Then all the records went through quality control procedures such as validating the taxonomy of the species by examining and re-identifying the specimens in museum collections and using taxonomic and geographic data quality control tools in the World Register of Marine Species (WoRMS) and the r-OBIS package (Provoost and Bosch 2017). This dataset can thus be further used for taxonomical and biogeographical studies of Solenidae.

## Introduction

Solenidae is an ecologically and economically important family of marine bivalves called razor clams (Cosel 1990, Saeedi et al. 2009, Saeedi et al. 2011, Saeedi et al. 2012). They comprise three genera, *Solen* Linnaeus, 1758 with 67 accepted species and *Solena* Mörch, 1853 with two accepted species, including *S. obliqua* and *S. rudis* and *Neosolen* (Ghosh, 1920), including species *N. aquaedulcioris* (Cosel 1989, Cosel 2002, Saeedi and Costello 2013, Saeedi et al. 2013, Bouchet 2014). These bivalves burrow down to about 30 cm in low intertidal and sub-tidal sandy – muddy sediments (Saeedi et al. 2009, Saeedi et al. 2013). They are suspension feeding bivalves with a free-swimming larva and adults can grow 20-30 mm per year in length (Saeedi et al. 2009). Solenidae are distributed worldwide in marine coastal and shelf ecosystems down to a depth of 100 m (Saeedi et al. 2013). The highest Solenidae species richness is in the Indo-Pacific and the family is absent from the polar regions and some oceanic islands such as New Zealand (Saeedi et al. 2013, Saeedi et al. 2016b).

Here, we publish a world dataset of Solenidae species distribution records that was compiled from our personal observations, literature, the Global Biodiversity Information Facility (GBIF) and the Ocean Biogeographic Information System (OBIS). A concern in using these data is that its fitness for use may be compromised by misapplication of species names, misspellings of names, occurrence of synonyms, errors in geo-referencing and mis-identification of specimens in museum collections (Costello et al. 2007, Robertson 2008, Saeedi and Costello 2013). Having an accurate and reliable geographic distribution dataset is necessary to study the global biodiversity and biogeography of the selected taxa. The present data have been vetted and used in several publications on species biogeography.

## General description

### Purpose

Publishing a standardised geographic distribution dataset that can be further used to study the taxonomy, biogeography, latitudinal species richness patterns and model future distributions of Solenidae.

## Sampling methods

### Study extent

Global

### Sampling description

Data on Solenidae species’ geographic distributions were obtained from the Global Biodiversity Information Facility (GBIF), Ocean Biogeographic Information System (OBIS), published literature, museum collections, personal correspondence and field sampling (). In addition, specimens of *Solen dactylus* were collected between 2006 to 2013 from Bandar Abbas in the south of Iran, northern Persian Gulf. Individuals were collected using a long steel wire with a V shape hook at one end) (Saeedi 2015). Specimens of *Solen marginatus* were collected using the salt method at A Pobra do Caraminal, A Coruna, Spain (42°36'47.6202" N, 8°55'38.5644" S) in February 2013 (Saeedi 2016).

### Quality control

We cross-referenced OBIS and GBIF data to avoid duplication of records. We excluded all records that were classified as fossils, were mapped on land and where location precision was unknown or more than 100 km. Regarding geographic data quality control, we used a gazetteer (http://www.marineregions.org/gazetteer.php?p=search) and r-OBIS R-package created by OBIS to quality control the data.

Regarding taxonomic quality control, all species’ names were verified in WoRMS (http://www.marinespecies.org/aphia.php?p=match) (Saeedi and Costello 2013, Bouchet 2014) and their synonyms and misspellings were reconciled.

**Figure 1.**
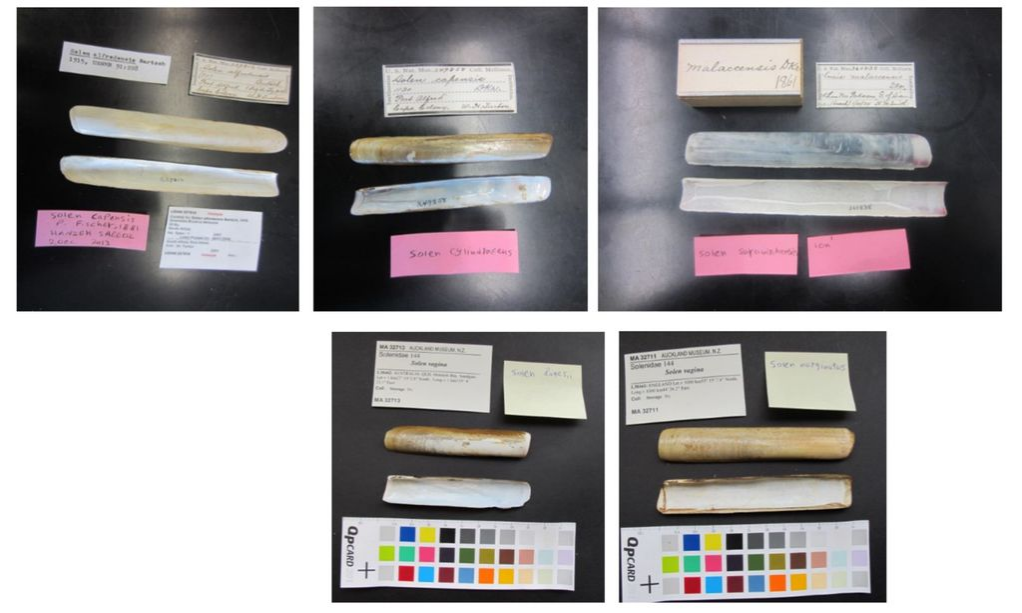
Mis-identifications of five species in Auckland and Smithsonian museums. About 35 species (150 specimens) in different museums were re-identified. The top three species were re-identified in Smithsonian (correct identification labels as pink sticker). The last two images show that *Solen fonesii* and *Solen marginatus* (correct identification labels as yellow sticker) were mis-identified as *Solen vagina*.

We also manually examined and re-identified specimens at the Natural History Museum of Paris (France), Auckland Museum (New Zealand), the National Museum of Natural History (Smithsonian) in Washington D.C. (USA) and the Natural History Museum of London (Fig. 1) and recorded where they had been collected.

## Geographic coverage

### Description

This study provides an integrated global geographic distribution dataset of all accepted Solenidae species.

## Taxonomic coverage

### Description

**Figure 2.**
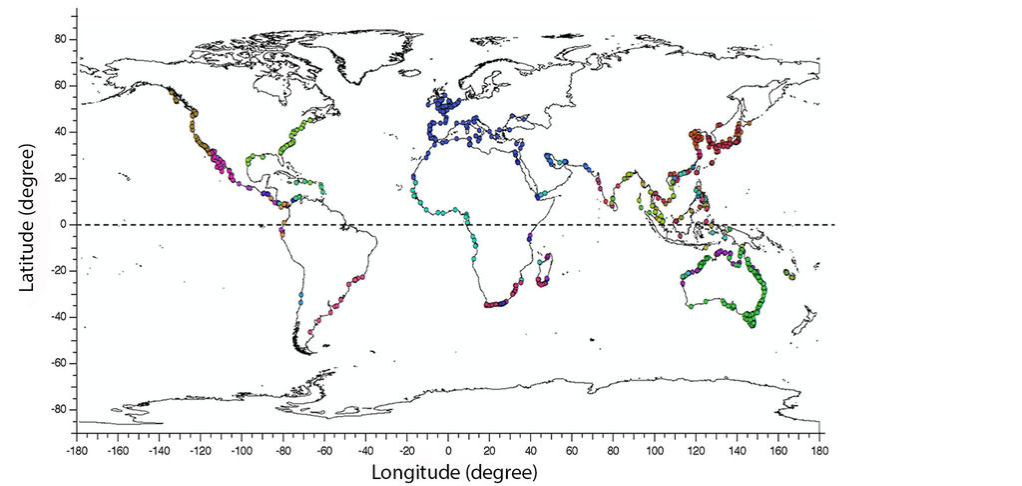
Global distribution of Solenidae species plotted using this dataset. Coloured circles show different species listed in Table 1. Dashed-line represents the Equator.

After the data-cleaning, a total of 3,034 *Solen* and *Solena* species records (Suppl. materials 1, 2, 3, 4) were collated and used to plot the global distributions of Solenidae species (Fig. 2). About 38% of distribution records (1,150 records) were extracted from GBIF and OBIS; about 58% of the records were collected from museum collections (1,733 records) and about 4% were sourced from literature and personal correspondence. *Solen marginatus* had the highest number of records, with about 20% of the total number of records of all Solenidae species. We found reliable coordinates for 67 of the 69 accepted *Solen* and *Solena* species, listed in WoRMS. We found another nine potential species described as aff. and cf. In total, 77 Solenidae taxa (we use the term 'taxa' as some species in Tables 1 and 2 were not identified to the species level) were used in this study (Suppl. material 1).

Solenidae species are distributed worldwide from -60 to +60 degree in latitude and they are absent from the polar regions and some oceanic islands such as New Zealand. In total, the eastern Pacific has 10 endemic *Solen* species and one endemic *Solena*, *S. rudis*. The Atlantic Ocean has eight *Solen* species and one endemic *Solena* species, *S. obliqua*, which occurs along the tropical mid-west Atlantic coasts of the Caribbean Sea. Europe and the Mediterranean Sea have only one species, *S. marginatus*, which is endemic. The Indo-West Pacific and north-west Pacific has the highest number of Solenidae species (about 50). New Caledonia has two *Solen* species that are both endemic.

### Taxa included

**Table:** 

Rank	Scientific Name	
kingdom	Animalia	
phylum	Mollusca	
kingdom	Bivalvia	
kingdom	Solenoidea	Razor clams
family	Solenidae	
genus	Solen	
genus	Solena	
genus	Neosolen	

## Temporal coverage

### Notes

1958 1 01 - 2013 2 01

## Usage rights

### Use license

Оpen Data Commons Open Database License (ODbL)

## Data resources

### Data package title

Global geographic distributions of Solenidae

### Number of data sets

1

### Data set 1.

#### Data set name

A world dataset on geographic distributions of Solenidae

#### Data format

Excel

#### Number of columns

31

#### 

**Data set 1.** 

Column label	Column description
eventID	An identifier created for each record as a combination of the source where the record was obtained and the available catalogue number where available. For example "urn:catalog:GBIF:Solenidae:20545" means that the record was obtained from GBIF with a catalogue number of 20545. Autonumber were used in the absence of a catalogue number.
eventDate	An identifier showing the date and time at which an occurrence was recorded where applicable.
minimumDepthInMeters	Minimum depth reported fro the record, in metres.
maximumDepthInMeters	Maximum depth reported for the record, in metres.
decimalLatitude	The geographic latitude (in decimal degrees, using the spatial reference system given in geodeticDatum) of the location where record was reported.
decimalLongitude	The geographic longitude (in decimal degrees, using the spatial reference system given in geodeticDatum) of the location where record was reported.
occurrenceID	A unique identifier for the occurrence record specifically created for this dataset.
scientificName	The accepted scientific name of the record.
scientificNameAuthorship	The accepted authorship of the scientific name.
scientificNameID	LSID number recorded for the accepted scientific name of the record.
kingdom	The full scientific name of the kingdom in which the taxon is classified.
taxonRank	The lowest taxonomic level where the record is identified.
identificationQualifier	Identification qualifications of the record for scientific names identified at the genus level (such as ?, confer or affinity).
occurrenceStatus	A statement about the presence or absence of the record reported from a location.
catalogNumber	Catalogue number of the record where available.
basisOfRecord	The nature of the record, i.e. whether the occurrence record is based on a stored specimen or an observation.
identificationReferences	A link to the original source where the record was obtained if available.
datasetName	The name identifying the dataset from which the record was derived.
institutionCode	The name (or acronym) in use by the institution having custody of the object(s) or information referred to in the record.
collectionCode	The name, acronym, coden or initialism identifying the collection or dataset from which the record was derived.
recordedBy	The names of people, groups or organisations responsible for recording the original occurrence.
dateIdentified	The date on which the subject was identified as representing the record.
identifiedBy	A list of names of people, groups or organisations who assigned the record to the subject.
countryCode	A unique identifier for the taxon represented in the row obtained from http://rs.gbif.org/vocabulary/iso/3166-1_alpha2.xml
locality	The specific description of the place where the record was reported.
locationAccordingTo	Information about the source of this location information. Here we mentioned the source where we extracted the data.
county	The full, unabbreviated name of the next smaller administrative region than country in which the location occurs.
stateProvince	The name of the next smaller administrative region than country in which the location occurs.
continent	The name of the continent in which the location occurs.
associatedMedia	A list of identifiers (e.g. publication, global unique identifier, URI) of media associated with the occurrence.
higherGeography	An identifier for the geographic region within which the Location occurred.

## Supplementary Material

Supplementary material 1 Geographic distribution of Solenidae and number of distribution point records (latitude and longitude) extracted from all databases. Data type: Geographic Distributions
Brief description: Numbers in the references indicate datasets extracted from GBIF and OBIS (Supplementary file 1). All other references cited in this table are listed in Supplementary file 2. WoRMS (World Register of Marine Species); MNHN (National Museum of Natural History); WAM (Western Australian Museum); QM (Queensland Museum); MV (Museum Victoria); AUSM (Australian Museum); AUCM (Auckland Museum); Smithsonian (Smithsonian Museum of Natural History); SBMNH (Santa Barbara Museum of Natural History).
File: oo_258680.docx Hanieh Saeedi and Mark J Costello

Supplementary material 2 A world dataset on geographic distributions of Solenidae Data type: Occurrences
File: oo_240562.xlsx Hanieh Saeedi and Mark J Costello

Supplementary material 3 Datasets used in this study from GBIF and OBIS, 2012-2014 Data type: Reference Dataset
Brief description: When similar data in both GBIF and OBIS were available, only OBIS data have been used
File: oo_118409.docx Hanieh Saeedi and Mark J Costello

Supplementary material 4 Literature list used to extract the distribution point records (latitude and longitude) of Solenidae species Data type: Reference Dataset
File: oo_118410.docx Hanieh Saeedi and Mark JCostello
