# Supplementary material for: A world dataset on the geographic distributions of Solenidae razor clams (Mollusca: Bivalvia)
Source: Biodivers Data J. 2019 Jan 31;(7):e31375. doi: 10.3897/BDJ.7.e31375 (PMC6367310; doi:10.3897/BDJ.7.e31375)
Supplement: Supplementary material 1 — Geographic distribution of Solenidae and number of distribution point records (latitude and longitude) extracted from all databases. [file bdj-07-e31375-s001.docx]

Geographic distribution of Solenidae and number of distribution point records (latitude and longitude) extracted from all databases. Numbers in the references indicate datasets extracted from GBIF and OBIS (Supplementary file 1). All other references cited in this table are listed in Supplementary file 2. WoRMS (World Register of Marine Species); MNHN (National Museum of Natural History); WAM (Western Australian Museum); QM (Queensland Museum); MV (Museum Victoria); AUSM (Australian Museum); AUCM (Auckland Museum); Smithsonian (Smithsonian Museum of Natural History); SBMNH (Santa Barbara Museum of Natural History).

| Species | GBIF | OBIS | Literature corres pondence | Museum | No. re cords | Location | Reference |
| --- | --- | --- | --- | --- | --- | --- | --- |
| Solen acinaces Hanley, 1843 |  |  | 1 |  | 1 | Tanzania | Personal contact with Rudo von Cosel |
| Solen acutangulus Dunker, 1868 |  | 1 |  |  | 1 | Mozambique [Morrumbene] | OBIS (23) |
| Solen aldridgei Nowell-Usticke, 1969 |  |  | 1 |  | 1 | Antigua island [Ramsay Bay] | WoRMS (Cosel, 2015) |
| Solen annandalei Preston, 1915 |  | 2 |  |  | 2 | India [Bay of Bengal; Mahanadi Estuary] | OBIS (20) |
| Solen aureomaculatus Habe, 1964 | 7 |  |  | 33 | 40 | Australia [Northern Australia: Gulf of Carpentaria, Groote Eylandt; Western Australia: Dampier Archipelago (East Lewis Island, off Enderby Island, southwest of Rocky Head, Dolphin Island, near east end of Searipple Passage, large bay on east coast, Georgeff Reef); Queensland: Townsville (Rowes Bay, Magnetic Is; Torres Strait, Great Barrier Reef, Cairncross Is, off Cape York Peninsula, Black beach); New South Wales: Kima] | GBIF (3);  MNHN; WAM; QM; MV; AUSM; AUCM; SBMNH |
| Solen brevissimusMartens, 1865 |  | 7 |  | 8 | 15 | Singapore [South Changi, SAF Changi Seaview]; China [Hong Kong] | OBIS (35);  MNHN; SBMNH |
| Solen canaliculatus Tchang & Hwang, 1964 |  | 12 |  | 33 | 45 | China; Taiwan [SW Tungkang] | OBIS (35);  MNHN |
| Solen cf. canaliculatus Tchang & Hwang, 1964 |  |  |  | 8 | 8 | China [NW Pratas Island, China Sea]; Philippines [Panabutan Pt., W Mindano]; Australia [Northern Territory: Charles Pt.] | Smithsonian |
| Solen capensis Fischer, 1881 |  | 14 | 1 | 30 | 45 | South Africa [Agulhas Bank, East of Martha Point, St Francis Bay, Lagoonal canal system at mouth of Kromrivier, St Lucia, Still bay, Sedgefield, Rietvlei, Jeffrey's bay, Port Elizabeth, Holes at foot of Lagoon Hermanus, Swartkops estuary, Yellow Sands, Nahoon estuary, Kwelera estuary, Knysna, Klein River Estuary, Hermanus, Port Alfred, Cape Colony, Capeland, Bushman's River, False Bay, Muizenberg, Plettenberg Bay, Zwartkops River]; Angola | OBIS (1, 15, 20, 23, 31); MNHN; Smithsonian; AUCM; SBMNH; Compton |
| Solen ceylonensisLeach, 1814 | 1 | 1 |  | 29 | 31 | India [Mandovi estuary,Goa; Konkan coast, Maharashtra; Bombay]; Kuwait; Yemen [Aden (Abyan beach, Khormaksar)]; South Africa [Tanzania (Zanibar)]; Ceylon (Sri Lanka); Indonesia; Suez, Read Sea | GBIF; OBIS (2, 20); MNHN; Smithsonian; AUCM |
| Solen corneus Lamarck, 1818 | 3 | 2 | 5 |  | 10 | Thailand [Nagasaki; Samut Songkhram; Samut Prakan Province; Don Hoi Lot]; Japan; South Korea; Philippines; Malaysia | GBIF; OBIS (2, 25); Cosel, 2002; Guerra et al., 2011 |
| Solen crockeri Hertlein & Strong, 1950 |  |  | 3 | 5 | 8 | Panama [Veraguas, Montijo District, Isla Gobernadora, NW, Darién]; El Salvador [La Paz, La Union]; Nicaragua [Golfo de Fonseca] | Keen, 1958; SBMNH |
| Solen cf. crosnieri Cosel, 1989 |  |  |  | 4 | 4 | Mozambique [Inhaka] | MNHN |
| Solen crosnieri Cosel, 1989 |  |  | 3 | 48 | 51 | Madagascar [Nosy Be; Tuléar (Taolagnaro; Tuléar Lagoon; Mahavatse; Nosy Lokaro, Anosy)] | Cosel, 1989; MNHN; WAM |
| Solen cylindraceus Hanley, 1843 |  | 5 | 3 | 123 | 131 | Mozambique [Androka; Ponta Pundvire, Inhaca Island]; Madagascar [Baie de sainte-Luce; Ranofotsy; Tuléar]; Tanzania [Mnazi Mmoja, Zanzibar City]; Yemen [Sheltered Bay, Bandar Tawahi, Aden]; Africa [30km northward to Djibouti, Somalia] | OBIS (20, 23, 31, 33); Paterson and Whitfield, 1997; MNHN; Smithsonian |
| Solen dactylus Cosel, 1989 |  |  | 10 | 36 | 46 | Saudi Arabia [Ras at Tanajib; Abu Ali Island]; Iran [Golshahr Coast, Bandar Abbas]; Kuwait [Kuwait Bay, NW coast of Kuwait, Persian Gulf]; Pakistan [Karachi; Baba Island] | Cosel, 1989; Saeedi et al., 2009-2013; MNHN; WAM; Smithsonian |
| Solen darwinensis Cosel, 2002 |  |  | 4 |  | 4 | Australia [Northern Queensland: North and westward to the tropical northern part of west Australia] | Cosel, 2002 |
| Solen delesserti Sowerby, 1874 | 1 | 5 |  | 32 | 38 | Madagascar [Anony; Tuléar; Nosy Be]; Mozambique [Maputo]; Singapore [South Changi, SAF Changi Seaview Resort; Ponggol Seventeenth Ave; Seletar; Off East Pulau Ubin; Changi sailing club, Changi]; India [Chilka Lagoon; Chilka Lake, Orissa; Cochin Harbour; Godavari estuary]; Philippines [Off Samal Island; vicinity of Cebu City, Cebu Island] ; Myanmar (Burma) [Tavoy]; South Africa [St Lucia Lake, northern Natal; Amatikulu River estuary; Inhambane, Durban; Mongue Ferry Morrumbene Estuary; Swartkops estuary; Kariega Estuary]; Djibouti; Yemen [Abyan beach, Aden] | GBIF; OBIS (2, 20, 37); MNHN; Smithsonian |
| Solen digitalis Jousseaume, 1891 |  |  |  | 14 | 14 | Yemen [Abyan beach; Khormaksar, Aden] | MNHN |
| Solen aff. exiguus Dunker, 1862 |  |  | 1 | 30 | 31 | Thailand [Kungkrabaen Bay, Gulf of Thailand]; Singapore [Off East Pulau Ubin, Changi sailing club, Red Cliff Shoal area, SAF Changi Seaview Resort, Changi Point Beach, Changi Greek, Changi beach park, Changi] | Simone, 2009; MNHN |
| Solen exiguus Dunker, 1862 |  |  |  | 2 | 2 | Philippines [Cebu] | Smithsonian |
| Solen cf. exiguus Dunker, 1862 |  |  | 1 |  | 1 | Hong Kong [Starfish Bay, Hoi Sing Wang] | Morton, 1988 |
| Solen fonesii Dunker, 1862 | 75 |  |  | 165 | 240 | Australia [Queensland: Dingo Bay; Broom (kimberely; near Dampier Creek;  north of Bush Point, Roebuck Bay); Brisbane (Moreton Bay: off Dunwich, Stradbroke Is; Nudgee Beach; Wynnum; SW Moreton bay, Manly; Sandgate); Hervey Bay (Dundowran Beach; Torquay; Pialba; Urangan; Great Sandy Strait, SE of Urangan); Keppel Bay (Yeppoon; Statute Point, N side and off NW end; Statute Point, N side; Kemps Beach; Zilzie Point, ca. 9 ml S of Yeppoon; Kemps Beach); Townsville (Rowes Bay; Bohle River mouth; Horseshoe bay, Magnetic Is, Horseshoe Bay; Pallarenda beach); Family Ids, National Park, Dunk Is; Cape York, Telegraph Station; GBR, Whitsunday Passage, Lindeman Is; Armstrongs Beach, Sarina; Lucinda; Seaforth; Town beach, Mackay, Boyne Island; Cannonvale Beach; Emu Creek, Sinclair Bay; Magnetic Island, Horseshoe Bay; Cardwell, Kennedy National Park; Brampton Reef , Bowen; Fraser Is, offshore of Sandy Point, S of Mooan; Burnett Heads; Near Woodstock, Cleveland Bay; Murdering Point to Big Maria Creek mouth; S of Innisfail, Kurrimine Beach); Western Australia: Port Headland; Eighty (Ninety) Mile Beach, Wallal Downs; Buccaneer Archipelago; Onslow area; Carnarvon; SE of Exmouth Homestead; 17ml S of Exmouth townsite, Exmouth Gulf; Melville Is, Cape Conder; Northern Territory: Gulf of Carpentaria (6 mls W of Van Diemens Inlet; beach between Tully Inlet & Massacre Inlet, ca. 30km E of NT border; 28mls S of Sweers Is; Groote Eylandt; beaches around Mapoon; NW and SW of Nassau River; 6 mls W of Van Diemens Inlet; beach between Tully Inlet & Massacre Inlet, ca. 30km E of NT border); Darwin (Tree Pt, Shoal Bay; Port Darwin; Lee Point; off Emery Point; Tree point, Shoal bay; Grose Is, WSW of Darwin); Machans Beach, North of Cairns; Cairns Harbour; Arnhem Land: Maningrida, beach below Crocodile Research Station; Boucaut Bay; Gove Peninsula, Yirrkala); New South Wales: Gloucester Is; Black beach, Kima] | GBIF (3, 42);  MNHN; WAM; QM; MV; AUSM; AUCM; Smithsonian; SBMNH |
| Solen gaudichaudi Chenu, 1843 |  |  | 2 |  | 2 | Chile [Coquimbo; Golf de Arauco] | Guerra et al., 2011 |
| Solen gemmelli Cosel, 1992 |  |  | 6 | 9 | 15 | Mexico [Sonora, Isla Candelero, El Golfo de Santa Clara, San Felipe, Ensenada Blanca, Magdalen Bay, La Paz, Santo Domingo, Baja California] | Cosel, 1992; SBMNH |
| Solen gordonis Yokoyama, 1920 | 10 |  |  | 29 | 39 | Japan [Miura Peninsula, Kanagawa; Akashi bay, Akashi; Kashima, Ibaraki Prefecture; Aichi, Mikawa; Wakayama, Minabe; Fukura, Awaji; Futami] | GBIF (2, 19, 38); MNHN; Smithsonian; AUCM; SBMNH |
| Solen cf. grandis Dunker, 1862 |  |  |  | 1 | 1 | Japan | Smithsonian |
| Solen grandis Dunker, 1862 | 13 | 19 | 6 | 12 | 50 | Japan [Mikawa; Sagami; Harima; Katase-Enoshima, Fujisawa, Kanagawa Prefecture; Ishikawa,Honshu Island; Aichi, Mikawa; Wakayama, Nada-Cho]; China [Laoshan Bay, Qingdao coast; Intertidal Zone Kimje; Shandong Peninsula, coastal regions of the Yellow Sea; Rizhao, Shandong Province; Beihai City, Beibu Gulf of Guangxi, southern China]; Republic of Korea [Buan-gun, Jeollabuk-do] | GBIF; OBIS (2, 18, 19, 30, 34, 35, 42, 44, 45, 46); Suh, 1993;  Xu and Song, 2008; Han et al., 2009;  Xu et al., 2011a, 2011b;  Yuan et al., 2011;  Zengqiong and Gangsheug, 2011;  Guerra et al., 2011; MNHN; Smithsonian; AUCM; SBMNH |
| Solen gravelyi Ghosh, 1920 |  | 1 |  |  | 1 | India [Chandipur] | OBIS (20) |
| Solen guinensisCosel, 1993 |  |  | 1 | 28 | 29 | Liberia [Monrovia]; Gabon [Cap esterias; Baie de Corisco]; Guinea [Dixinn port, Conakry]‎; Nigeria ‎[Okobaba sawmills along the Lagos lagoon]; Mauritania [Nouakchott]; Senegal [Cap Skirring, Ziguinchor]; Senegal [Dakar]; Ghana; Côte d'Ivoire; Cameroon; Equatorial Guinea; Congo; Angola | MNHN; Smithsonian; Cosel, 1993 |
| Solen jonesii Hanley, 1842 |  |  |  | 1 | 1 | Philippines [Isla de Cebú] | Personal contact |
| Solen kajiyamai Habe, 1964 | 8 |  |  | 18 | 26 | Australia [Western Australia: Onslow Jetty, Onslow; Dolphin Island; north section of east coast; north of Nelson Rocks, Dampier Archipelago; Queensland: Dingo beach; Cape York "township"; North Keppel Is, off NW end Keppel Bay; Yeppoon, Keppel Bay; Turkey beach, between Bundaberg and Gladstone; Karumba; beaches around Mapoon; Sweers Is, Gulf of Carpentaria] | GBIF (3); MNHN; WAM; Smithsonian; AUSM; AUCM |
| Solen kempi Preston, 1915 |  | 2 |  |  | 2 | India [Chilka Lake, Orissa; Sundarban] | OBIS (20) |
| Solen kikuchii Cosel, 2002 |  |  | 1 | 2 | 3 | Japan [ Nagato, Konagai, Kita-Takagi, Nagasaki] | Cosel, 2002; MNHN |
| Solen krusensterni Schrenck, 1867 | 14 |  |  | 16 | 30 | Japan [Hakodate; Kitaibaraki, Ibaraki Prefecture; Kashima city, Ibaraki Prefecture; Wakayama, Nada-Cho; Otaru, Hokkaido Prefecture; Miyako Bay, Iwate-ken; Rikuzen; Kurihama; Futtsu]; Russia [Primorskiy Krai, Sakhalin, Zeleny Island] | GBIF (2, 19, 34, 45); MNHN; Smithsonian; SBMNH |
| Solen kurodai Habe, 1964 | 6 | 1 |  | 25 | 32 | Japan [Kushi, Minamiboso, Chiba Prefecture; Aichi, Mikawa; Kushimoto; Suiken; Kamakura; Wakayama; Tanabe, Kii Strait]; Australia [Queensland: Shelly beach, Townsville; Keppel Bay; Sarina beach, S Mackay] | GBIF; OBIS (1, 2, 34, 45); MNHN; AUCM |
| Solen cf. lamarckii Chenu, 1843 |  |  |  | 30 | 30 | Philippines [Luzon (Quezon, Tayabas Bay, Sariaya); Cadiz (Negros Occidental); Mindanao (Misamis Oriental, Villanueva)]; New Caledonia [Grand Récif; Balabio; Coral Sea; Poum] | MNHN |
| Solen lamarckii Chenu, 1843 | 1 | 3 |  |  | 4 | India [Tamil Nadu; Bay of Bengal; Krusadai Island; Parangipettai]; Indonesia [Myanmar] | OBIS (20) |
| Solen leanus Dunker, 1862 | 1 |  |  | 4 | 5 | Philippines [Cebu]; Malaysia [Sandakan]‎ | GBIF (2); Smithsonian |
| Solen linearis Spengler, 1794 |  | 1 | 3 | 1 | 5 | Singapore [Red Cliff Bank, Changi]; Nicobar Islands | GBIF; OBIS (2, 35);  Cosel, 2002 |
| Solen lischkeanusDunker, 1865 |  |  |  | 10 | 10 | Egypt [Suez] | MNHN |
| Solen madagascariensis Cosel, 1989 |  |  | 2 | 5 | 7 | Madagascar  [Tuléar Lagoon; Nosy Be; SW of Port, Shell concentrates at LT, Tulear] | Cosel, 1989; MNHN; WAM |
| Solen malaccensis Dunker, 1862 |  |  | 1 | 13 | 14 | Thailand [Samut Songkhram]; Philippines [Aurora, Baler Bay, Baler; Quezon City, Tayabas Bay, Sariaya, Luzon; Negros Occidental, Cadiz] | Guerra et al., 2011; MNHN |
| Solen marginatus Pulteney, 1799 | 352 | 169 | 41 | 80 | 642 | France [Arcachon Bay; Golfe de Porto-Vecchio, Corsica; Bretagne; Côtes-d'Armor;  Berck]; Spain [Atlantic coast of Huelva; Barra de Cangas; Pontevedra, Boiro; Ría de Arousa; Regueira; Asturias; Vila Real de Santo Antonio; Galicia;  Ria de Rebadeo; Santander Bay; Cantabria; Sotavento; Melilla; A Pobra do Caraminal, A Coruna;  Andalucia, Cádiz;  Plazas de Soberania]; Egypt [Port Said]; UK [Chichester Harbour; Porth Dinllaen; Lleyn Peninsula; Milford Haven, Pembrokeshire; Tenby, Pembrokeshire; Hampshire;  Wales, Conwy]‎; Tunisia [12 k N Raoued; Gammarth; Maltin Wadi in Sfax]; Belgium [West Vlaanderen]; Morocco [Bou Regreg estuary; Mellila]; Italy [Adria bei Monfalcone; Istrien; Angle Bay; Ferrara; Porto Garibaldi; Forli; Cesenatico; La Eo ria; Rimini;  Noto; Veneto; Lido di Venezia, Venezia; Emilia-Romagna]; Turkey [Kocaeli; Izmit Korfezi]; Cyprus; Greece [Kavalla; Thasos; Alyki, Zakynthos; Laganas; Saronikos; Saronikos];  Russia [Krasnodar Krai];Ukraine [Odessa]; Portugal [Averio; Faro; Ria Formosa lagoon;  Setúbal district]; Israel [Haifa];  Lebanon [Akkar];  Netherlands; Belgium; Ireland; Algeria;  Mauritania;  Malta;  Albania | GBIF; OBIS (2, 5, 6, 7, 12, 14, 21, 22, 24, 28, 29, 30, 31, 39, 40, 41, 51, 53); Cosel, 1993; Sauriau et al., 1989;  Thompson et al., 1999;  Vale and Sampayo, 2002; Neves and Mano, 2005;  Remacha-Triviño , 2005; López and Darriba, 2006;  Remacha-Triviño and Anadón, 2006; López-Flores et al., 2008;  da Costa and Martínez-Patiño, 2009;  da Costa et al., 2008;  Rufino et al., 2010; Guerra et al., 2011; MNHN; Smithsonian; AUCM |
| Solen mexicanus Dall, 1899 | 1 |  |  | 1 | 2 | Mexico [Golfo de Tehuantepec]; Nicaragua ‎[San Juan del Sur] | GBIF (2); Smithsonian |
| Solen moolenbeeki Thach, 2014 |  |  | 1 |  | 1 | Vietnam [NHA Trang, Khanh Hoa Province] | WoRMS (Thach, 2014) |
| Solen oerstedii Mörch, 1860 |  |  | 1 |  | 1 | Costa Rica | WoRMS (Coan and Valentich-Scott, 2012) |
| Solen pazensis Lowe, 1935 | 1 |  | 1 | 10 | 12 | United States [Digg's Point, California]; Mexico [La Paz;  Nayarit, Santa Cruz to Platanitos,  Baja California] | Keen, 1958; GBIF (2); AUCM; SBMNH |
| Solen pfeifferi Dunker, 1862 |  |  | 2 | 32 | 34 | Peru [Tumbez]; Panama [Farfan beach, Canal Zone; East and West End of Venado Beach; Isla Venado; Playa Venado; Coclé;  Isla Contadora; Fort Amador, Canal Zone];  Mexico [Guerrero, Bahia Zihuatanejo; Nayarit; Jalisco; Colima; Sinaloa, Bahia Topolobampo; Sonora, Isla Alcatraz; Sinaloa, Playa Las Gaviotas, Mazatlan; Oaxaca, La Paz; Ensenada Blanca, San Felipe, Baja California]; Ecuador [Guayas, Punta Jacinto] | Keen, 1958; Personal contact; Smithsonian; SBMNH |
| Solen pseudolinearis Cosel, 2002 |  |  | 2 |  | 2 | China | Cosel, 2002 |
| Solen regularis Dunker, 1862 |  |  | 4 |  | 4 | Malaysia [Asajaya Laut and Buntal; Serpan; Moyan, Sarawak]; Thailand [Don Hoi Lord, Upper Gulf of Thailand, Province of Samut Songkhram, 100km west of Bangkok] | Worrapimphong et al., 2010; Rinyod and Rahim, 2011 |
| Solen rosaceus Carpenter, 1864 | 5 |  | 2 | 55 | 62 | United States [La Paz; Long Beach; Terminal Island, San Pedro Bay; Sunset Beach; Santa Barbara; Vacation Isle, San Diego County; Terminal Island; Alamitos Bay; Venice Beach, Los Angeles County; Orange County, California]; Mexico [Sonora; San Felipe; Mazatlan, Sinaloa; Agua Chale; Estero Percebu, Baja California] | Keen, 1958; Cosel, 2002; GBIF (2); QM; MV; Smithsonian; AUCM; SBMNH |
| Solen roseomaculatus Pilsbry, 1901 | 1 | 4 |  | 55 | 60 | Yemen [Abyan beach, Aden]; Madagascar [Tuléar; 35 mi W Cap St Andro; 25 mi NW of Cap St Andre]; Mozambique [Inhaca, Portuguese Island; 80 mi SSW Ponta da Barra; At Linga-Linga Morrumbene in channel N of wrecked schooner]; Iran [Bandargah, Kish, The Persian Gulf]; Japan [Takehara, Hiroshima; Hirado, Hizen; Okinawa; Nagasaki]; Indonesia [Off West Coast of wasir Island, W Wocam, Aru]; Philippines [Marin Bay, Mindoro]; Ceram [Off Tutuhuhur, Piru Bay] | GBIF; OBIS (2, 23, 26, 35); MNHN; Smithsonian |
| Solen rosewateri Altena, 1971 |  | 8 |  |  | 8 | Colombia [Costa Verde; Bahia Portete; Pozos Colorados; Rodadero] | OBIS (26) |
| Solen rostriformis Dunker, 1862 | 2 | 10 | 21 | 43 | 76 | Peru [Tumbez]; Panama [Darién]; Mexico [Guerroro Negro; Santo Domingo; Sonora, Isla Candelero; Jalisco; Nayarit, San Blas; Harbor Breakwater, La Paz; Mazatlan, Sinaloa; Puerto, Penusco, San Carlos; Estero Percebu; Bahia Concepcion; San Felipe; Magdalen Bay; Peninsula de El Mogote; Punta Banda, Baja California]; United States [La Paz Bay; Mission Bay; San Pedro; La Bay; Newport Bay; Morro Bay; Santa Barbara; Orange County; San Diego County, Burma, California; San Pedro, Los Angeles] | GBIF; OBIS (16, 12);  Coan and Valentich-Scott, 2012; Cosel, 1992; SBMNH |
| Solen sarawakensis Cosel, 2002 | 2 | 1 | 18 |  | 21 | Malaysia [N of Kuching, Buntal Beach; Morib, Selangor; Sandakan; Bintulu, Sarawak] | GBIF (11, 50); Cosel, 2002; MNHN; Smithsonian |
| Solen schultzeanus Dunker, 1850 |  |  |  | 1 | 1 | Australia [Queensland:  Brisbane (Moreton Bay)] | QM |
| Solen sicarius Gould, 1850 | 3 | 47 | 1 | 52 | 103 | Mexico [Bahía San Quintín, Ensenada, Baja California]; United States [Los Angeles County; Orange County; Monterey County; Los Angeles County; Angel Island, Marin County; San Diego County; Ventura County; Balboa Island, Orange County; Terminal Island, Los Angeles County; San Luis Obispo County; Santa Barbara County; Monterey County; San Luis Obispo County, Morro Bay; Ventura County; Long Beach; Cabrillo Beach, California; Oregon, Curry County; Alaska, Kuiu Island, Wrangell-Petersburg County; Mason County; Olympia; Puget Sound; Victoria; King County; San Juan County, Turn Island, Washington]; Canada [Naden Harbor; Graham Island; Virago Sound; Tasu Sound; W of Moresby Island; Sandspit Bar, Deadtree Point; Naden Harbour; Skidegate] | GBIF; OBIS (2, 16, 17, 48);  Coan et al., 2012; Smithsonian; SBMNH |
| Solen cf. sloanii Gray, 1843 |  |  |  | 25 | 25 | New Caledonia [Koumac] | MNHN |
| Solen sloanii Gray, 1843 | 3 | 8 |  | 48 | 59 | Japan [Ryukyu Islands; Akashi Harima; Amamura, Awaji; 1km WNW Onna Vilage (Horseshoe Cliffs), Okinawa]; Mozambique [80 mi SSW Ponta da Barra; Inhaca]; Madagascar [Tuléar; Tanjon' Andavaka; Taolagnaro; Marolinta]; South Africa [Kosi Bay off; SE of Sheffield Beach; SE of Umhlanga Beach; Cape St. Lucia off]; Indonesia [Lembeh-Strait, NE Sulawesi]; Vietnam [Nha Trang] | GBIF; OBIS (33, 35); MNHN; Smithsonian; SBMNH |
| Solen soleneae Cosel, 2002 |  |  | 2 | 14 | 16 | Japan [Awase, Okinawa, Okinawa Prefecture]; Philippines [Marin Bay, Mindoro]; Malaysia; China | Cosel, 2002; MNHN; Smithsonian |
| Solen cf. strictus Gould, 1861 |  |  |  | 2 | 2 | Japan  [Goga, Nago, Okinawa Prefecture] | MNHN |
| Solen strictus Gould, 1861 | 74 | 3 | 3 | 54 | 134 | Japan [Enoshima; Chiba ; Nagasaki; Hakodate; Honmoku, Hirado, Hizen; Tokyo Bay, Honshu; Takasago; Yamaguchi, Yamaguchi Prefecture; Nago City, Okinawa Prefecture; TSU city, Mie prefecture beach; Takou; Aomori; Aichi, Mikawa;  Kanagawa; Mie, Tsu-shi; Kochi, Shikoku Island, Wakayama]; China [Cheffo; Qingdao (Tsingtao)]; Malaysia [Sarawak]; Korea  [Kushima, Ariake Bay (North Pacific Ocean); Cheju-do; Simpo tideland, Outfal Estuary of Kumkang River, West of Korea]; Russia [Primorskiy Krai] | GBIF; OBIS (12, 18, 19, 25, 33, 45);  Hong and Lee, 1990;  Xu et al., 1999; Kim et al., 2004; MNHN; Smithsonian; AUCM; SBMNH |
| Solen tairona Cosel, 1985 | 1 | 1 | 2 |  | 4 | Costa Rica [Gandoca, Frente al puesto del MINAE; N Costa Verde]; Colombia [Cartagena, Crespo; Santa Marta] | GBIF; OBIS (26); Cosel, 1985 |
| Solen aff. tchangi Huber, 2010 |  |  |  | 1 | 1 | Philippines [Negros Occidental] | MNHN |
| Solen tchangi Huber, 2010 |  |  |  | 23 | 23 | Philippines [Negros Occidental, Cadiz] | MNHN |
| Solen thachi Cosel, 2002 | 1 |  |  | 4 | 5 | Vietnam [Long Hai; Dong Hai; Khanh Hoa, Nha Trang | GBIF (31); MNHN |
| Solen thailandicus Cosel, 2002 |  |  | 2 | 12 | 14 | Thailand [Don Hoi Lot]; Vietnam [Hai Tien; Bang Poo] | Cosel, 2002;  Guerra et al., 2011; MNHN; Smithsonian |
| Solen thuelchus Hanley, 1842 | 1 | 20 | 1 | 7 | 29 | Brazil [Codo Beach; Rasa da Cotinga Island; Santana's Archipelago; Rio Grande do Sul; Praia do Camaroeiro; Praia Pereque;  Rio de Janeiro]; Argentina [Mar Del Plata, Northern Argentina; Desembocadura Río Negro;  Chubut]; Uruguay [Costa de Maldonado; Rocha] | GBIF; OBIS (8, 9, 10, 13, 27, 47); PenchasZadeh et al., 2006; AUCM; SBMNH |
| Solen timorensis Dunker, 1852 |  |  | 2 |  | 2 | Indonesia [Timor]; Papua New Guinea | WoRMS (Cosel, 2015) |
| Solen vagina Linnaeus, 1758 | 1 | 3 | 1 | 33 | 38 | Malaysia [Kuantan estuary, Tanjung Lumpur, Mangrove area, Phang; George Town, Penang]; India [Calcutta; Digha Coast]; Vietnam [Nha Trang; Chu Lei Bay, Da Nang]; Singapore [Changi beach park, Changi]; Philippines [Negros Occidental, Cadiz]; Thailand [Andaman Sea, Krabi, Ban Laem]; Myanmar (Burma) [Maungmagan; Tavoy]; Andaman & Nicobar Islands | GBIF; OBIS (2, 20); MNHN; Smithsonan; AUCM |
| Solen vaginoides Lamarck, 1818 | 137 |  |  | 294 | 431 | Australia [Queensland: Brisbane (20 km N of Brisbane, Redcliffe beaches; off Dunwich, Stradbroke Is; Stradbroke Is, Dunwich; Redcliffe; 2mls SE of Redlands Bay jetty; off Peel Is, Moreton Bay); Keppel Bay (Zilzie Point, ca. 9 ml S of Yeppoon; North Keppel Is, off NW end; off Humpy Is); Townsville (Rowes Bay; magnetic island, Horseshoe Bay, off Townsville); Great Barrier Reef (E of Sarina, Mackay; E of Broad Sound; Swain Reefs, 3km NE of W side of Bylund (Gillett) Cay; off Cairns; W of Hayman Is; Lizard Island, Watsons Bay; Whitsunday Passage, Lindeman Is); Wonga Beach, S end, N of Mossman; Sandy Strait State School, Urangan; Caloundra; offshore of Sandy Point, S of Mooan, Fraser Is; Tannum Sands, S of Gladstone; Pialba; Shelly Beach, Torquay, Hervey Bay; Port Curtis, off Gatcombe Head; Lucinda; Cape Flattery; ; Torres Strait, Friday Is; Bowen; Disaster Bay; Dingo beach; Telegraph Station; Somerset Bay, Cape York; Kurrimine Beach, S of Innisfail; Mission Beach, near Tully; Flat Rock, North Richmond River entrance; Rudder Reef 30 miles NE of Mossman; Amity point, N. stradbroke Is; Long Beach, Joskeleigh; New South Wales: Sydney (Port Stephens, Hawks Nest Beach; Cremorne, W side of Frederick Henry Bay; Sow & Pigs Reef; Middle Harbour,The Spit, Port Jackson; Manly Beach; Port Hacking, Gunnamatta Bay; off entrance to Botany Bay; Bate Bay, Cronulla Beach; Georges River, Dolls Point to Sans Souci baths; Botany Bay, from Cooks River to Kyeemagh Baths; Quarantine Bay, North Head, Sydney Harbour; off SE end Chinamans Beach; Balmoral, off Wy-ar-gine Pt (=Wyargine Pt); Balmoral, N end Edwards Beach; N side of Middle Harbour, Castle Rock, Middle Harbour; Pittwater; off Patonga Beach; Ettalong Beach, Broken Bay); Victoria (Corner inlet; Apollo Bay; Western Port Bay; Port Welshpool; Philip Island); Botany Bay; Twofold Bay; Jervis Bay; Eden; Newtons Beach, Nadgee Fauna Reserve; Merimbula, off Surfshed; Off Nelson Bay, Port Stephens; In Great Oyster Bay; Seven Mile Beach, W side of Frederick Henry Bay; Mallacoota; Bundeena beach; Gloucester Is; Broulee, S of Batemans Bay; Eastern Bass Strait, 3.2 km S of Cape Conran; St francis island, Nuyts Arch; Cave bay, Hunter Island, off NW tip; 13.3 km E of eastern edge of Lake Tyers; 15.3 km ESE of eastern edge of Lake Tyers; 20.2 km WSW of Pt Ricardo; 5.4 km SW of Cape Conran, Eastern Bass Strait; Crib Point, Western Port, CPBS station 32N; Between Stony Point and Crib Point; Off Cheseapeake, French Island; Chinamans beach; Port Phillip Bay, Sorrento pier; Tuncurry, Wallis Lake; Southern Australia: Tasmania (Wineglass Bay, Freycinet Peninsula; D'Entrecasteaux Channel, 1ml W of Simpsons Point; Eaglehawk Neck, N end Pirates Bay; Swansea; Port Arthur; Stanley; Greens Beach; Binalong Bay; Bridport; Coles Bay; Kelso; Ulverstone; Browns River; Kingston; Cambridge; Wineglass Bay; Rheban; King Island; Flinders Island; Bakers beach; Seven Mile Beach, Hobart; Swansea; Black Rock Beach); Adelaide (Gulf St. Vincent, Largs Bay; Semaphore Beach; Largs Beach; Grange; Port Willunga); Melbourne (Waratah Bay; Flinders and Shoreham); Kangaroo Is, Hog Bay; Port Lincoln; Port Noarlunga; Cowes, Phillip Island; Great Australian Bight, 75ml E of Rocky Point; Northern Territory: (beaches around Mapoon; Groote Eylandt, Gulf of Carpentaria); Western Australia: Shoalwater Bay, off Canoe Passage between Townshend & Marquis Ids; Swan Is, W side, Shoalwater; Duke Group, Marble Is, Homestead Bay; Albany | GBIF (2, 3, 20, 36, 42); MNHN; AUCM; QM; MV; AUSM; Smithsonian; SBMNH |
| Solen viridis Say, 1821 | 5 | 61 | 1 | 30 | 97 | United States [Sullivan's Bay, South Carolina; Newport, Rhode Island; Galveston Island;  San Patricio, Texas; Smith Island; Holden Beach, New Carolina; Simone Island; Newport, Rhode Island; Georgia; Delaware Bay;  Horry County; Hunting Island; Charleston bay, South Carolina; Beaufort Harbour, North Carolina; Atlantic City; Off 5th Saint & Beach, Wildwood; South Cape May beach; Anglesea; Egg Harbor, New Jersey; Hog I, Philadelphia; Jekyll Island; near Turner's Creek, Wilimington River; one mile offshore of S end of Ossabaw Isl, Georgia; Bay Marchand Lease Area, 500-2000 Ms N Of Platform] | GBIF; OBIS (2, 4, 16, 32, 34, 36, 37, 49, 52);  Williams and Porter, 1971; Smithsonian; AUCM; SBMNH |
| Solen vitreus Dunker, 1862 |  |  |  | 1 | 1 | Malacca | WoRMS (Cosel, 2015) |
| Solen woodwardi Dunker, 1862 |  |  |  | 11 | 11 | Philippines [Negros Occidental, Cadiz] | MNHN |
| Solena obliqua (Spengler, 1794) | 3 | 2 |  | 4 | 9 | Panama [Chiriqui lagoon]; Haiti [Les Cayes]; Venezuela [Manaure]; Colombia [Bahia de Nenguange]; Saint Lucia [north coast]; Dominican Republic [Monti Cristi, Santo Domingo] | GBIF; OBIS (2, 12, 26); Smithsonian |
| Solena rudis (C.B. Adams, 1852) | 5 |  | 3 | 21 | 29 | Panama [Punta Batele, near Pacific entrance of canal; Panama City; Farfán; Chame; Panama Bay; Venado Beach;  West Panama, Punta Bique, Arraijan District; Playa Venado; Isla Venado]; Brazil [Vera Cruz (Camarones)]  ; Ecuador [Esmeraldas];  Peru; Costa Rica | GBIF (2, 12, 26);  Coan and Valentich-Scott, 2012; Keen, 1958; Smithsonian; AUCM; SBMNH |
| Total | 738 | 413 | 163 | 1717 | 3034 | No of Countries > 65 | |
