# Supplementary material for: A world dataset on the geographic distributions of Solenidae razor clams (Mollusca: Bivalvia)
Source: Biodivers Data J. 2019 Jan 31;(7):e31375. doi: 10.3897/BDJ.7.e31375 (PMC6367310; doi:10.3897/BDJ.7.e31375)
Supplement: Supplementary material 3 — Datasets used in this study from GBIF and OBIS, 2012-2014 [file bdj-07-e31375-s003.docx]

| **NO** | **Dataset** | **Citation where provided** | **GBIF** | **OBIS** | **No. species** |
| --- | --- | --- | --- | --- | --- |
| 1 | Academy of Natural Sciences OBIS Mollusc Database | Rosenberg *et al*., 2002, Ocean Biogeographic Information System: Academy of Natural Sciences OBIS Mollusc Database. Accessed via http://www.gbif.org/dataset/83a09216-f762-11e1-a439-00145eb45e9a on 2013-10-15 | × | × | 2 |
| 2 | Academy of Natural Sciences (MAL) | … | × |  | 19 |
| 3 | Australian Museum provider for OZCAM | … | × |  | 4 |
| 4 | Biodiversity of the Gulf of Mexico Database (BioGoMx) | Moretzsohn, F., J. Brenner, P. Michaud, J.W. Tunnell, and T. Biodiversity of the Gulf of Mexico Database (BioGoMx). January 1, 2010. Harte Research Institute for Gulf of Mexico Studies (HRI), Texas A&M University-Corpus Christi (TAMUCC), Corpus Christi, Texas. Version: 1.0 Data Presentation Form: Database Online Resource: http://www.iobis.org/ |  | × | 1 |
| 5 | Biogeographic data from BODC - British Oceanographic Data Centre | British Oceanographic Data Centre, UK. Biogeographic data from BODC. in : EurOBIS. http://www.marbef.org/data/eurobissearch.php?dataprovider=47, accessed on [15/ 10/ 2013]. |  | × | 1 |
| 6 | Biologiezentrum Linz | … | × |  | 1 |
| 7 | BioMar (EurOBIS) | Picton, B.E., C.S. Emblow, C.C. Morrow, E.M. Sides, P. Tierney, D. McGrath, G. McGeough, M. McCrea,P. Dinneen, J. Falvey, S. Dempsey, J. Dowse, and M. J. Costello, 1999: Marine sites, habitats and species data collected during the BioMar survey of Ireland. Environmental Sciences Unit, Trinity College, Dublin, Ireland | × | × | 1 |
| 8 | Brazilian Marine Invertebrate Data Sets from SpeciesLink | … |  | × | 1 |
| 9 | Centro de Estudos do Mar - CEM, UFPR | … |  | × | 1 |
| 10 | Colección Nacional de Invertebrados - Museo Argentino de Ciencias Naturales 'Bernardino Rivadavia' | … | × |  | 2 |
| 11 | Collection Mollusca SMF | … | × |  | 1 |
| 12 | The Conchological Society of Great Britain & Ireland - Mollusc (marine) data for Great Britain and Ireland | … | × |  | 1 |
| 13 | Corbisier 1991 1994 Benthic Macrofauna | … |  | × | 1 |
| 14 | Countryside Council for Wales (CCW) Technical Support (Research & Monitoring) Contracts, Wales | … | × |  | 1 |
| 15 | East London Museum | East London Museum - Mollusc Collection |  | × | 2 |
| 16 | EPA'S EMAP Database | U.S. Environmental Protection Agency Environmental Monitoring and Assessment Program (EMAP), http://www.epa.gov/emap/." | × | × | 3 |
| 17 | Gwaii Haanas Invertebrates (OBIS Canada) | Living marine legacy of Gwaii Haanas. II: Marine invertebrate baseline to 2000 and invertebrate-related management issues. | × | × | 1 |
| 18 | Gyeryonsan Natural History Museum Shellfish | … | × |  | 2 |
| 19 | Ibaraki Nature Museum, Mollusk collection | … | × |  | 4 |
| 20 | IndOBIS, Indian Ocean Node of OBIS | Chavan, VIshwas and C. T. Achuthankutty (editors), IndOBIS Catalogue of Life, Available at http://www.indobis.org/, Retrived 15/ 10/ 2013 |  | × | 10 |
| 21 | Inventaire national du Patrimoine naturel (INPN) | … | × |  | 1 |
| 22 | Invertebrates (GBIF-SE:SMNH, Swedish Museum of Natural History) | … | × |  | 1 |
| 23 | Iziko South African Museum - Mollusc Collection (AfrOBIS) | iziko South African Museum - Mollusc Collection | × | × | 4 |
| 24 | Joint Nature Conservation Committee - Marine Nature Conservation Review (MNCR) and associated benthic marine data held and managed by JNCC | Ostler, R. Marine Nature Conservation Review (MNCR) and associated benthic marine data held and managed by JNCC. Joint Nature Conservation Committee, Centre for Ecology and hydrology, Aberdeenshire, UK. | × | × | 1 |
| 25 | KOBIS database | … |  | × | 2 |
| 26 | Marine and Coastal Research Institute - INVEMAR, Colombia, IABIN | INVEMAR. SIBM en línea: Sistema de Información sobre Biodiversidad Marina. Santa Marta: Instituto de investigaciones Marinas y Costeras José Benito Vives de Andréis,. http://www.invemar.org.co/siam/sibm/index.htm |  | × | 3 |
| 27 | Marine invertebrate from Argentina, Uruguay and Chile | Bigatti, G. (2010). Marine invertebrate from Argentina, Uruguay and Chile. Centro Nacional Patagónico, Puerto Madryn, Chubut, Argentina. Retrieved from http://www.iobis.org. |  | × | 1 |
| 28 | Marine Life List of Ireland | Allen D., Beckett B., Brophy J., Costello M.J., Emblow C., Maciejewska B., McCrea M., Nash R., Penk M. & Tierney A. Marine species recorded in Ireland during field suveys by EcoServe, Ecological Consultancy Services Ltd. Available online at http://www.marbef.org/data/eurobis.php. Consulted on 2013-10-15 |  | × | 1 |
| 29 | Merseyside BioBank - North Merseyside General Record sets | … | × |  | 1 |
| 30 | Museum Victoria provider for OZCAM | … | × |  | 1 |
| 31 | Natal Museum - Mollusc Collection (AfrOBIS) | Natal Museum - Mollusc Collection | × |  | 1 |
| 32 | National Benthic Inventory (NBI) | http://www.nbi.noaa.gov | × | × | 2 |
| 33 | Natural Geography In Shore Areas (NaGISA) Dataset | Rigby,P.R., B.Konar, T.Kato, K.Iken, H.Chenelot and Y.Shirayama (2005)NaGISA OBIS Dataset ver.1 | × | × | 4 |
| 34 | Natural History Museum Rotterdam | … | × |  | 11 |
| 35 | NCOS1959_Mollusca (OBIS China) | … | × | × | 7 |
| 36 | NEFSC Benthic Database | Northeast Fisheries Science Center, National Marine Fisheries Service, NOAA, U.S. Department of Commerce. 2010. NEFSC Benthic Database. Northeast Fisheries Science Center, 166 Water Street, Woods Hole Laboratories, Woods Hole, MA 02543.Retrieved from http://www.usgs.gov/obis-usa/ |  | × | 1 |
| 37 | NMNH Invertebrate Zoology Collections | Department of Invertebrate Zoology, Research and Collections Information System, NMNH, Smithsonian Institution. See: http://www.mnh.si.edu/rc/db/collection_db_policy1.html | × | × | 2 |
| 38 | NOAA HML Tidal Creek Database | Tidal Creek Database, NOAA Oceans and Human Health Initiative, NOAA Hollings Marine Laboratory | × | × | 1 |
| 39 | North Sea Benthos Survey | Craeymeersh J., P. Kingston, E. Rachor, G. Duineveld, Carlo Heip, Edward Vanden Berghe, 1986: North Sea Benthos Survey. |  | × | 1 |
| 40 | Peabody Invertebrate Zoology DiGIR Service (IP) | … | × |  | 1 |
| 41 | Pembrokeshire Marine Species Atlas | Dale Rostron. Pembrokeshire Marine Species Atlas. Countryside Council for Wales, Gwynedd, UK. |  | × | 1 |
| 42 | Queen Victoria Museum Art Gallery provider for OZCAM | … | × |  | 3 |
| 43 | Sea shell specimens database of Mr. Toru Ueda collection, Shizuoka Prefecture Museum of Natural History | … | × |  | 1 |
| 44 | Shellfish specimens in The Goulandris Natural History Museum (GNHM-MS) | … | × |  | 1 |
| 45 | Shellfish specimens in the NISHINOMIYA SHELL MUSEUM (NSMK-MS) | … | × |  | 2 |
| 46 | Shellfish Specimens of Miyazaki Prefecture Museum of Nature and History | … | × |  | 1 |
| 47 | SINBIOTA - marine data (OBIS South America, BRAZIL) | Marine Benthos - BIOTA/FAPESP | × | × | 1 |
| 48 | SOMBASE | … | × |  | 1 |
| 49 | South TX Outer Continental Shelf and MI, AL, and FL Outer Continental Shelf benthic organism sampling 1974-1978 | US National Oceanographic Data Center. 2011. South TX Outer Continental Shelf and MI, AL, and FL Outer Continental Shelf benthic organism sampling 1974-1978. US National Oceanographic Data Center, Silver Spring, Maryland, USA.Retrieved from http://www.usgs.gov/obis-usa/. |  | × | 1 |
| 50 | SysTax | … | × |  | 1 |
| 51 | Taxonomic Information System for the Belgian coastal area (EurOBIS) | Flanders Marine Institute (VLIZ). Taxonomic Information System for the Belgian coastal area. 10 Aug 2004, Oostende, Belgium, Accessed on [15/10/2013]. | × | × | 1 |
| 52 | The Southeast Regional Taxonomic Center (SERTC) Invertebrate Database: Invertebrates of the Southeastern United States | Marine Resources Research Institute, South Carolina DNR | × | × | 1 |
| 53 | Zoological Museum Amsterdam, University of Amsterdam (NL) – Mollusca_Netherlands | … | × |  | 1 |
