# Supplementary material for: A world dataset on the geographic distributions of Solenidae razor clams (Mollusca: Bivalvia)
Source: Biodivers Data J. 2019 Jan 31;(7):e31375. doi: 10.3897/BDJ.7.e31375 (PMC6367310; doi:10.3897/BDJ.7.e31375)
Supplement: Supplementary material 4 — Literature list used to extract the distribution point records (latitude and longitude) of Solenidae species [file bdj-07-e31375-s004.docx]

**REFERENCES**

Coan, E.V., Valentich-Scott, P. Frank & R. Bernard (2012). Bivalve Seashells of Western North America: Marine bivalve molusks from Arctic Alaska to Baja California. Santa Barbara Museum of Natural History. Santa Barbara Musuem of; 1st edition (May 2000). 764 pp.

Coan, E.V. & Valentich-Scott, P. (2012). Bivalve seashells of tropical west America: Marine bivalve mollusks from Baja California to Peru. Santa Barbara Museum of Natural History. Santa Barbara.1258 pp, 326 pls.

Compton, J. S. (2006). The mid-Holocene sea-level highstand at Bogenfels Pan on the southwest coast of Namibia. *Quaternary Research*, **66**(2), 303-310.

Cosel, R. v. (1985). A new Solen from the caribbean coast of Colombia, South America (Bivalvia, Solenidae). *Bulletin of the Museum National d’Histoire Naturelle, Paris, series A*, **7**(2): 328–333.

Cosel, R. v. (1992). “*Solen rosaceus*” - three species. *Veliger,* **35**, 366–380.

Cosel, R. v. (1993). The razor shells of the eastern Atlantic. Part 1: Solenidae and Pharidae I (Bivalvia: Solenacea). *Archiv für Molluskenkunde*, p. 207 – 321.

Cosel, R. v. (2015). *Solen aldridgei* Nowell-Usticke, 1969. In: MolluscaBase (2015). Accessed through: World Register of Marine Species at http://www.marinespecies.org/aphia.php?p=taxdetails&id=413337 on 2015-07-22

Cosel, R. v. (2015). *Solen timorensis* Dunker, 1852. In: MolluscaBase (2015). Accessed through: World Register of Marine Species at http://www.marinespecies.org/aphia.php?p=taxdetails&id=413759 on 2015-07-22

Cosel, R. v. (2015). *Solen vitreus* Dunker, 1862. In: MolluscaBase (2015). Accessed through: World Register of Marine Species at http://www.marinespecies.org/aphia.php?p=taxdetails&id=413525 on 2015-07-22

da Costa, F., & Martínez-Patiño, D. (2009). Culture potential of the razor clam *Solen marginatus* (Pennánt, 1777). *Aquaculture,* **288**(1-2), 57-64.

da Costa, F., Darriba, S., & Martínez-Patiño, D. (2008). Embryonic and larval development of *Ensis arcuatus* (Jeffreys, 1865) (Bivalvia: Pharidae). *Journal of Molluscan Studies,* **74**(2), 103-109.

Guerra, A., Lodeiros, C., Gaspar, M.B. & da Costa, F. (2011). Razor clams: biology, aquaculture and fisheries. Xunta de Galicia, Consellería do Mar, Santiago de Compostela, Spain. ISBN: 978-84-453-4986 1 (Hard cover book). ISBN: 978-84-453-4987-8 (CD).

Han, E. T., Whang, J. D., & Chai, J. Y. (2009). *Himasthla alincia* (Echinostomatidae): Metacercariae in brackish water bivalves and their growth and development in experimental animals. *Journal of Parasitology,* **95**(6), 1415-1420.

Hong, S. S., & Lee, J. J. (1990). Histological studies on the gametogenesis and the reproductive cycle of razor clam, *Solen strictus* Gould, in Cheju-do. *Bulletin of the Marine Resources Research Institute, Jeju National University.* **14**, 39-59.

Keen, A. M. (1971). Sea Shells of Tropical West America: Marine Mollusks from Baja California to Peru: Stanford University Press. 624 pp.

Kim, Y. G., Yu, J. E., Chung, E. Y., & Chung, P. R. (2004). *Acanthoparyphium tyosenense* (Digenea: Echinostomatidae): Experimental confirmation of the cercaria and its complete life history in Korea. *Journal of Parasitology,* **90**(1), 97-102.

López, C., & Darriba, S. (2006). Presence of *Marteilia* sp. (Paramyxea) in the razor clam *Solen marginatus* (Pennánt, 1777) in Galicia (NW Spain). *Journal of Invertebrate Pathology,* **92**(2), 97-99.

López-Flores, I., Garrido-Ramos, M. A., de la Herran, R., Ruiz-Rejón, C., Ruiz-Rejón, M., & Navas, J. I. (2008). Identification of *Marteilia refringens* infecting the razor clam *Solen marginatus* by PCR and in situ hybridization. *Molecular and Cellular Probes,* **22**(3), 151-155.

Morton, B. (1988). The anterior pallial tentacles of *Solen* aff. *exiguus* (Bivalvia: Solenacea) from Hong Kong. *Journal of Molluscan Studies,* **54**(1), 135-137.

Neves, N. M., & Mano, J. F. (2005). Structure/mechanical behavior relationships in crossed-lamellar sea shells. *Materials Science and Engineering C,* **25**(2), 113-118.

Penchaszadeh, P. E., Arrighetti, F., Cledón, M., Livore, J. P., Botto, F., & Iribarne, O. O. (2006). Bivalve contribution to shallow sandy bottom food web off Mar Del Plata (Argentina): inference from stomach contents and stable isotope analysis. *Journal of Shellfish Research,* **25**(1), 51-54.

Remacha- Triviño, A., & Anadón, N. (2006). Reproductive cycle of the razor clam *Solen marginatus* (Pulteney 1799) in Spain: A comparative study in three different locations. *Journal of Shellfish Research,* **25**(3), 869-876.

Remacha-Triviño, A. (2005). Density, biometry and sex ratio of the razor clam *Solen marginatus* (Pulteney, 1799) in Eo ria (northwestern Spain). *Boletín - Instituto Español de Oceanografía,* **21**(1-4), 387-394.

Rinyod, A. M. R., & Rahim, S. A. K. A. (2011). Reproductive cycle of the razor clam *Solen regularis* Dunker, 1862 in the western part of Sarawak, Malaysia, based on gonadal condition index. *Journal of Sustainability Science and Management,* **6**(1), 10-18.

Rufino, M. M., Gaspar, M. B., Pereira, A. M., Maynou, F., & Monteiro, C. C. (2010). Ecology of megabenthic bivalve communities from sandy beaches on the south coast of Portugal. *Scientia Marina,* **74**(1), 163-178.

Saeedi, H., Ardalan, A. A., Kiabi, H. B., & Zibaseresht, R. (2012). Metal concentrations in razor clam *Solen dactylus* (Von Cosel, 1989) (Bivalvia: Solenidae), sediments and water in Golshahr coast of Bandar Abbas, Persian Gulf. *Iranian Journal of Fisheries Science,* **11**(1), 165-183.

Saeedi, H., Raad, S. P., Ardalan, A. A., Kamrani, E., & Kiabi, B. H. (2009). Growth and reproduction of *Solen dactylus* (Bivalvia: Solenidae) on northern coast of the Persian Gulf (Iran). *Journal of the Marine Biological Association of the United Kingdom,* **89**(8), 1635-1642.

Sauriau, P. G., Mourei, V., & Rincé, J. P. (1989). Trophic system of wild soft-bottom mollusks in the marennes-oleron oyster-farming bay. *Oceanologica Acta,* **12**(2), 193-204.

Simone, L. R. L. (2009). Anatomical description of *Solen* cf. *exiguus* Dunker from Thailand: (Bivalvia: Solenidae). *Archiv f&#252;r Molluskenkunde: International Journal of Malacology,* **138**(2), 113-122.

Suh, H. L. (1993). *Anthessius kimjensis*, a new species of Anthessiidae (Copepoda; Poecilostomatoida) associated with the pelecypod *Solen grandis* Dunker in Korea. *Hydrobiologia,* **259**(3), 187-193.

Thach N.N. (2014). A new Solen from Vietnam (Bivalvia: Solenidae). *Miscellanea* *Malacologica*. **6**(4): 61-64.

Thompson, S., Budzinski, P., Garrigues, H., & Narbonne, J. F. (1999). Comparison of PCB and DDT distribution between water-column and sediment-dwelling bivalves in Arcachon Bay, France. *Marine Pollution Bulletin, 38*(8), 655–662.

Vale, P., & De M. Sampayo, M. A. (2002). Esterification of DSP toxins by Portuguese bivalves from the Northwest coast determined by LC-MS - A widespread phenomenon. *Toxicon,* **40**(1), 33-42.

Williams, A. B., & Porter, H. J. (1971). A ten-year study of meroplankton in North Carolina estuaries: Occurrence of postmetamorphal bivalves. *Chesapeake Science,* **12**(1), 26-32.

Worrapimphong, K., Gajaseni, N., Le Page, C., & Bousquet, F. (2010). A companion modeling approach applied to fishery management. *Environmental Modelling and Software,* **25**(11), 1334-1344.

Xu, K., & Song, W. (2008). Two trichodinid ectoparasites from marine molluscs in the Yellow Sea, off China, with the description of *Trichodina caecellae* n. sp. (Protozoa: Ciliophora: Peritrichia). *Systematic Parasitology,* **69**(1), 1-11.

Xu, K., Kanno, M., Yu, H., Li, Q., & Kijima, A. (2011a). Complete mitochondrial DNA sequence and phylogenetic analysis of Zhikong scallop *Chlamys farreri* (Bivalvia: Pectinidae). *Molecular Biology Reports,* **38**(5), 3067-3074.

Xu, K., Lei, Y., Al-Rasheid, K. A. S., & Song, W. (2011b). Two new ectoparasitic ciliates, *Sphenophrya solinis* sp. nov. and *Planeticovorticella paradoxa* sp. nov. (Protozoa: Ciliophora), from marine molluscs. *Journal of the Marine Biological Association of the United Kingdom,* **91**(2 SPEC. ISSUE), 265-274.

Xu, K., Song, W., & Warren, A. (1999). Trichodinid ectoparasites (Ciliophora: Peritrichida) from the gills of mariculture molluscs in China, with the descriptions of four new species of *Trichodina Ehrenberg*, 1838. *Systematic Parasitology,* **42**(3), 229-237.

Yuan, L., Sun, L., Wei, G., Long, N., Xie, Z., & Wang, Y. (2011). 9,400 yr B.P.: The mortality of mollusk shell (Mya truncata) at high Arctic is associated with a sudden cooling event. *Environmental Earth Sciences,* **63**(6), 1385-1393.

Zengqiong, H., & Gangsheng, Z. (2011). A new structural model of bivalve ligament from *Solen grandis*. *Micron,* **42**(7), 706-711.
